# Trajectories of psychiatric care in an innovative outpatient program designed for transitional age youth (16 to 24 years old) in French-speaking Belgium: results of a retrospective study

**DOI:** 10.1192/j.eurpsy.2022.288

**Published:** 2022-09-01

**Authors:** S. Marchini, J. Reis, I. Hussein, V. Delvenne

**Affiliations:** 1 Queen Fabiola Children’s University Hospital, Child And Adolescent Psychiatry, Brussels, Belgium; 2 Erasme Hospital, Child And Adolescent Psychiatry, Brussels, Belgium; 3 Service de Santé Mentale à l’ULB, Child And Adolescent Psychiatry, Brussels, Belgium; 4 Brugmann University Hospital, Adult Psychiatry And Medical Psychology, Brussels, Belgium

**Keywords:** AMHS, mental health, transitional age youth, CAMHS

## Abstract

**Introduction:**

Transitional age youth (TAY), from 16 to 24 years old, are a particularly at-risk population in mental health. They have specific needs, not currently covered between child and adolescent mental health services (CAMHS) and adult mental health services (AMHS), mainly because of existing barriers.

**Objectives:**

This retrospective study was carried out to describe sociodemographic and clinical characteristics of 243 patients who attended a new TAY-tailored outpatient psychiatric program.

**Methods:**

Outcomes related to trajectories of psychiatric care were analysed, such as leading symptom, consultation’s referral and requester, and final orientation.

**Results:**

The sample was mainly composed by female; the average age was 18.7 (± 2.0) years. Leading symptoms were divided into three dimensions: internalizing (67.5%), externalizing (21.8%) and psychotic (10.7%). Leading symptom differed according to sex (p<0.001), with internalizing symptoms more frequent in women, externalizing and psychotic symptoms more frequent in men. Patients presenting psychotic symptoms were significantly older than both those with internalizing (p=0.016) and externalizing symptoms (p=0.008). After first assessment, 81.5% of youth were followed-up in our specific outpatient program, without any difference according to sex (p=0.081) or leading symptom (p=0.092). Overall and final psychiatric orientation are showed in the flowchart.

**Figure:**
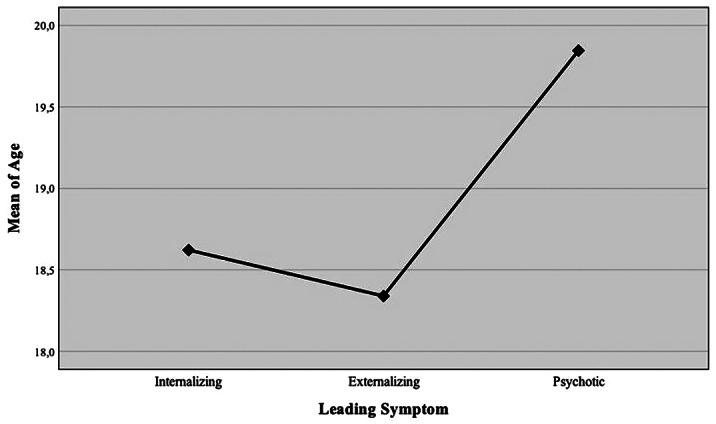

**Figure:**
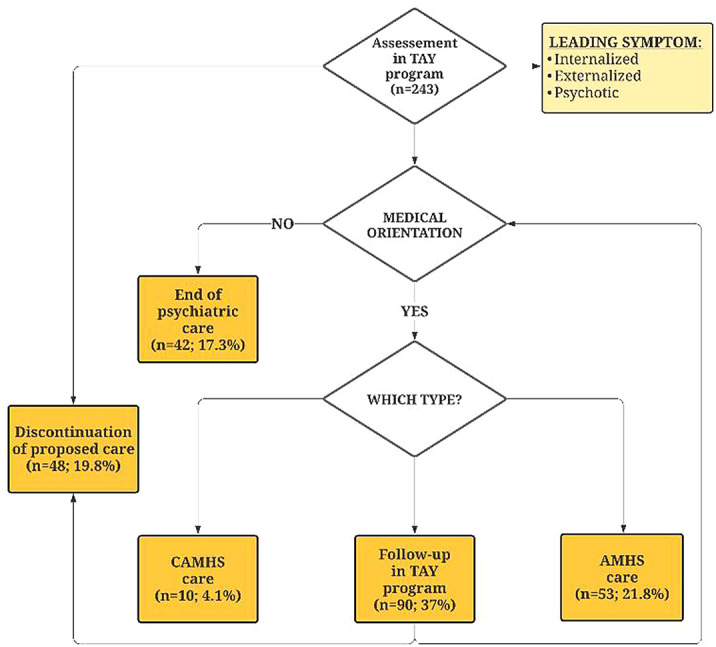

**Conclusions:**

This TAY-tailored psychiatric outpatient program represents an innovative contribution to reinforce CAMHS-AMHS interface in French-speaking Belgium. This study enlightens the importance to enhance clinical expertise in youth mental health. Classical boundaries, determined by artificial variables such as age or type of psychopathology, do not seem to be efficient criteria to achieve a good quality psychiatric evaluation and continuity of care in TAY.

**Disclosure:**

The authors declare no potential conflicts of interest. The study was carried out as part of the University Chair “Psychiatry in Transition in a World in Transition” (Université Libre de Bruxelles - ULB) with the support of Julie Renson Fund, the Queen Fa

